# Bringing the Pediatric Endocrine Spanish Speaking Community Together: First Virtual Pediatric Endocrine Meeting in Low- and Middle-Income Countries in Central and South America

**DOI:** 10.2196/41353

**Published:** 2023-05-08

**Authors:** Roberto Bogarin, Luis Elizondo, Evangelia Kalaitzoglou, Jadranka Popovic, Alan Rogol, Erick Richmond, Jean-Pierre Chanoine, Jose M Lopez-Pedrosa, Francis Ruiz Salazar, Patricia Vuguin

**Affiliations:** 1 Costa Rica National Children´s Hospital San Jose Costa Rica; 2 Hospital Calderon Guardia San Jose Costa Rica; 3 University of Kentucky Lexington, KY United States; 4 Pediatric Alliance Allegheny Health Network Pittsburgh, PA United States; 5 University of Virginia Charlottesville, VA United States; 6 University of British Columbia Vancouver, BC Canada; 7 Abbott Nutrition Research and Development Abbott Laboratories Granada Spain; 8 Hospital Mexico San Jose Costa Rica; 9 Columbia University New York, NY United States

**Keywords:** continuing medical education, continuing education, medical education, professional development, pediatric, child, endocrinology, endocrine, pediatric endocrinology, diabetes, low- and middle-income countries, Latin America, Spanish, virtual, resources, digital

## Abstract

**Background:**

Pediatric endocrinology is a specialty that is struggling worldwide to maintain adequately trained professionals. Pediatric endocrine care in Central America and Caribbean countries is often performed by pediatricians or adult endocrinologists due to the limited number of pediatric endocrinologists. These health care providers are seldom members of endocrine societies and frequently lack formal training in the field.

**Objective:**

In this study, we describe the scope of a virtual conference in pediatric endocrinology and diabetes targeted to low- and middle-income countries to provide equal opportunities for access to medical education for health care professionals.

**Methods:**

The virtual conference was sponsored by the Pediatric Endocrine Society (North America), Asociación Costarricense de Endocrinología (previously, Asociación Nacional Pro Estudio de la Diabetes, Endocrinología y Metabolismo), and Asociacion Centroamericana y del Caribe de Endocrinologia Pediátrica. The conference was free to participants and comprised 23 sessions that were either synchronous with ability for real-time interactive sessions or asynchronous sessions, where content was available online to access at their convenience. Topics included idiopathic short stature, polycystic ovarian syndrome, diabetes mellitus, telemedicine, Turner syndrome, congenital adrenal hyperplasia, obesity, central precocious puberty, and subclinical hypothyroidism. The participants were asked to evaluate the conference after its completion with a questionnaire.

**Results:**

A total of 8 speakers from Spain, Canada, Costa Rica, and the United States delivered the virtual event to 668 health care professionals from Guatemala, Venezuela, Dominican Republic, Costa Rica, Ecuador, Peru, Uruguay, Mexico, Honduras, Argentina, the United States, Bolivia, Chile, Panama, El Salvador, Nicaragua, Paraguay, Belize, Spain, and Colombia. Name, profession, and country were fully disclosed by 410 (61.4%) of the 668 health care professionals. The profession or level of training of participants were as follows: pediatric endocrinologists (n=129, 19.3%), pediatricians (n=116, 17.4%), general practitioners (n=77, 11.5%), adult endocrinologists (n=34, 5.1%), medical students (n=23, 3.4%), residents in various specialties (n=14, 2.1%), and others (n=17, 2.6%). A total of 23 sessions were offered, most of which were bilingual (Spanish and English). Feedback from the evaluation questionnaire indicated that the content of the conference was very relevant to the participants’ professional practice. Additionally, the participants reported that they were very satisfied with the organization, the web-based platform, and the sessions of the conference.

**Conclusions:**

Lack of accessibility to the latest and cutting-edge medical education in pediatric endocrinology and diabetes for medical professionals from low- and middle-income countries can be overcome with a virtual conference. Online availability, low cost, and easy-to-use technology were well received from the participants, who were overall very satisfied by the quality and the relevance of the sessions to their professional practice.

## Introduction

Pediatric endocrinology is a field that is faced with increased demand, partly due to an increase in the burden of obesity and diabetes [1]. Interestingly, one-fifth of individuals with type 1 diabetes are in low- and lower-middle–income countries [2]. Although there is a high demand for pediatric endocrinologists to meet clinical care needs, even in developed countries, there is concern about the future of the pediatric endocrinology workforce due to a diminished interest in the specialty [3]. Furthermore, global inequality in pediatric endocrine care has been well recognized and may be in part due to the lack of access to a formal clinical training in pediatric endocrinology in developing countries [4].

An initiative to address these disparities included a free and globally accessible multilingual e-learning website for pediatric endocrinology and diabetes [5] supported by the European Society for Paediatric Endocrinology [6]. The website provides health care professionals access to topics in pediatric endocrinology and diabetes aimed to improve their clinical skills and competencies through interactive learning. In addition to self-directed learning, it can serve as a valuable resource that can facilitate classroom teaching and promote interaction with experts in the field [7]. A section of the website, targeted toward health care professionals from resource-limited settings, is available in 5 languages, including Spanish [6]. Another initiative to overcome the global inequality to access to medical knowledge and training has been the creation of a Paediatric Endocrinology Training Center for Africa, which resulted in 54 fellows from 12 countries being trained in pediatric endocrinology and diabetes [8]. Additional initiatives have been implemented in Sudan to improve pediatric endocrinology services with the establishment of a local pediatric endocrinology training program [9].

Pediatric endocrine care in Central America and the Caribbean countries is often provided by general pediatricians or adult endocrinologists due to lack of trained pediatric endocrinologists. General pediatricians are rarely Pediatric Endocrine Society (PES) members or members of other local pediatric endocrine organizations. Additionally, many of them are not able to participate in such conferences due to financial limitations. Specifically, as outlined by Pulido et al [10], medical education in Latin America has been characterized by marked differences due to variable socioeconomic and cultural factors. Moreover, the countries with the lowest density of human resources in health in the Americas are all in Central America (Guatemala and Honduras), South America (Bolivia and Guyana), and the Caribbean (Haiti) [11]. Previous conferences with primary focus on pediatric endocrine care were organized by the International Relations Committee from the PES and were held in person, one in Costa Rica in 2014 and one in the Dominican Republic in 2019. The conference in Costa Rica was partly supported by PES and attracted more than 50 attendees from Central America with focus on the educational needs of the local pediatricians and pediatric endocrinologists. In 2019, a second conference was organized by PES and Sociedad Domincana de Pediatria Endocrinologica, which was well attended (150 attendees from the Dominican Republic and other Central American countries).

Due to challenges associated with the COVID-19 pandemic, holding another in-person conference was not feasible. Instead, a virtual conference was held from August 31 to September 21, 2020 (Multimedia Appendices 1 and 2). The goals of this virtual meeting were to generate a new approach to encourage interactions between clinicians interested in pediatric endocrinology and diabetes and to improve medical knowledge in this field. This initiative was developed by the PES and local organizations to support a group of underrepresented and diverse health care professionals (clinicians, researchers, and educators) from Spanish and non–Spanish speaking countries with the goal to educate and promote health and eventually, reduce pediatric endocrine health inequalities in Central America and in the Caribbean. This outreach program has provided education to local providers focused on endocrine-related conditions in underserved areas.

## Methods

### Overview

A 3-week conference (1er Congreso Virtual Asociación Centroamericana de Endocrino Pediátrica), sponsored by the Pediatric Endocrine Society (North America), Asociación Costarricense de Endocrinología (previously Asociación Nacional Pro Estudio de la Diabetes, Endocrinología y Metabolismo) and Asociación Centroamericana y del Caribe de Endocrinología Pediátrica—the Central American and Caribbean Association of Endocrinology, Diabetes and Metabolism—took place from August 31 to September 21, 2020. The speakers were pediatric endocrinologists from the United States, Canada, Spain, and Costa Rica, and most were members of the PES. During the conference, which consisted of a hybrid model of synchronous and asynchronous sessions, a total of 23 sessions were delivered to a diverse audience. Topics included idiopathic short stature, polycystic ovarian syndrome, diabetes, telemedicine, Turner syndrome, congenital adrenal hyperplasia, obesity, precocious puberty, and subclinical hypothyroidism (Table 1).

**Table 1:** Agenda of the conference, as well as synchropnous (S) or asynchronous (A) format.

Number	Session title	S or A
1	Catch up growth in stunted children	A
2	Idiopathic short stature	A
3	Practical Implementation of telemedicine	S
4	Subclinical hypothyroidism	A
5	Catch up growth in stunted children: Meet the expert	S
6	What should I do with an obese patient?	S
7	Idiopathis short stature: Meet the expert	S
8	Subclinical Hypothyroidism: Meet the expert	S
9	Turner Syndrome	A
10	Precocious puberty: Controversies in management	A
11	Growth Hormone indications	S
12	Congenital Adrenal Hyperplasia	A
13	What would I do with a patient with PCOS?	S
14	Turner Syndrome: Meet the expert	S
15	Congenital Adrenal Hyperplasia: Meet the expert	S
16	Challenges managing the adolescent with diabetes	A
17	Insulin pumps: Update/What’s new	A
18	Liraglutide in Children and Adolescents with Type 2 diabetes	S
19	Pubertal delay: Approach and treatment	A
20	What would I do with a patient with gynecomastia?	S
21	How do I approach a patient with secondary amenorrhea?	S
22	Diabetes: Meet the expert	S
23	Pubertal delay: Meet the expert	S

Registration was free for participants and attendees registered online [12]. The participants had access to live sessions using GoTo Webinar, a landing page at endopediatrica’s website [12], or a Moodle website to access the lectures either synchronously or asynchronously. The asynchronous sessions (Vimeo; Vimeo, Inc.) included content that was available online and could be accessed by the participants at their convenience. The designated speaker made a presentation in the asynchronous prerecorded sessions with a defined theme. The audience had the opportunity to interact with this speaker for a live or synchronous session 3-5 days later. These sessions were in “question & answer,” “clinical cases,” or “ask the expert” format. Live sessions were broadcasted using Facebook live (Meta Platforms, Inc.) and Vimeo with opportunities to participate in interactive sessions with Q&A format. Attendees obtained information and technological tools to access the meeting from multiple sources (Facebook, WhatsApp [Meta Platforms, Inc.], GoTo Webinar, Zoom [Zoom Video Communications], and Vimeo), which allowed for the participation of individuals beyond the Central America and Dominican Republic regions. At the end of the meeting, the participants were invited to complete an evaluation of the conference. Conference evaluations included questions about the relevance of the topics covered, registration process, satisfaction with technological platforms, access to the event, and the invited speakers' knowledge of the subjects and presentations.

### Ethical Considerations

No ethics approval was applied for because the conference-reported data are anonymous or deidentified and therefore exempt from oversight. There was no process of data linkage and recording, and dissemination did not generate identifiable information. No monetary compensation was offered to the speakers or participants. The meeting received generous support from Novo Nordisk, Pfizer, and Merck Foundation to cover the expenses of the English-to-Spanish translation, the production of the platform, and the promotion of the event. Topics were chosen by the organizers based on the needs and interest of the audience. Novo Nordisk, Pfizer, or Merck did not provide topics for discussion.

## Results

**Figure 1:**
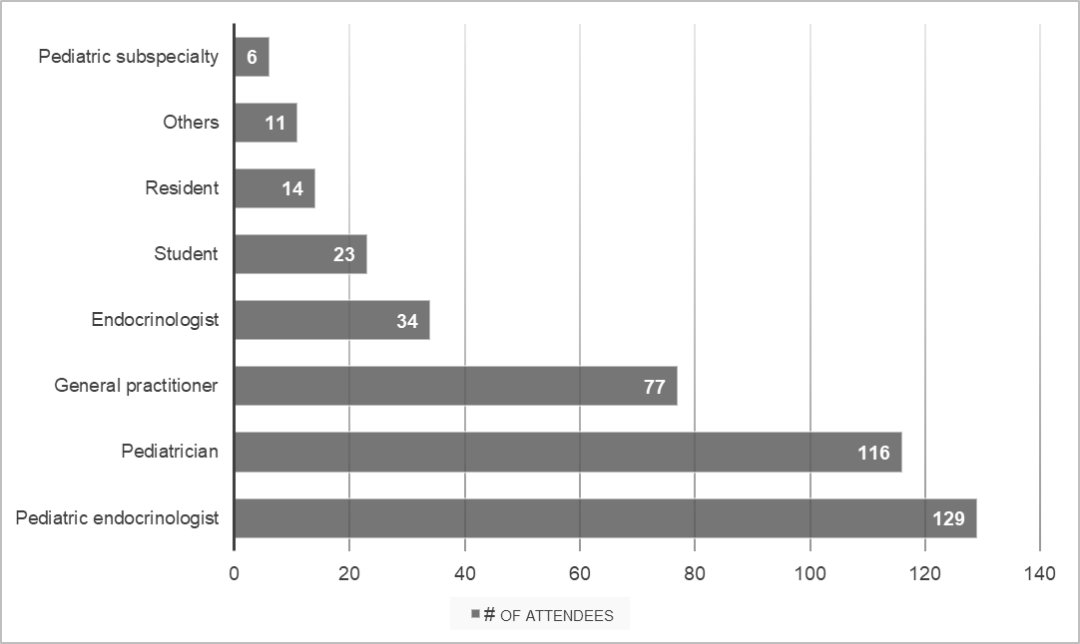
Profession and/or level of training of attendees. Information available for 410 attendees.

A total of 8 speakers from Spain, Canada, Costa Rica, and the United States delivered the virtual event to 668 health care professionals. The audience consisted mainly of native Spanish speakers, and Spanish was the preferred language at the conference. Full registration information was available for most of the participants, including their country of origin and their profession or level of training. The country with the most attendees was Guatemala (n=69, 10.3%), followed by Venezuela (n=66, 9.9%), and Dominican Republic (n=63, 9.4%; Table 2). Participants were from 20 countries (Guatemala, Venezuela, Dominican Republic, Costa Rica, Ecuador, Peru, Uruguay, Mexico, Honduras, Argentina, the United States, Bolivia, Chile, Panama, El Salvador, Nicaragua, Paraguay, Belize, Spain, and Colombia). The majority of the participants were pediatric endocrinologists, pediatricians, or general practitioners (Figure 1), with residents and medical students also participating.

Of the 23 sessions, 14 (61%) sessions were live (Table 1), and if presented by an English speaker, they had simultaneous Spanish translation. Using the GoTo Webinar platform, 360 records of access were documented for the live sessions. Meet-the-expert and live sessions, which consisted of questions and answers as well as interaction with the audience showed an average interest by the audience of 92%, and a total number of 168 questions were asked throughout the event. Access to the lectures post meeting was evaluated via the Vimeo platform during September 2020. Of the 2937 times a video was played, 1285 (43.8%) times the platform reported 100% completion of the video. Vimeo reported 1331 total hours of content playback. The number of people who viewed a prerecorded session (Vimeo) on a particular day ranged from 41 to 253.

**Figure 2:**
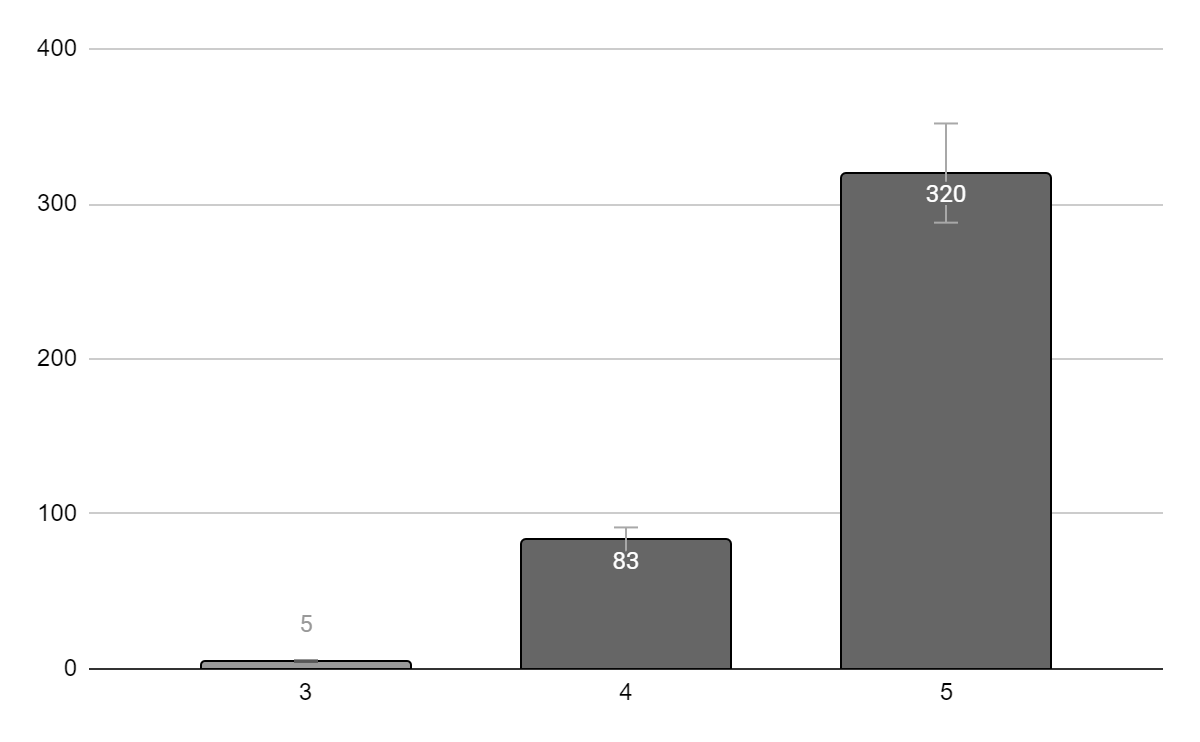
"How satisfied were you with the event?" Evaluation of the conference by the attendees. Information available for 408 attendees (1=not satisfied to 5=very satisfied).

**Figure 3:**
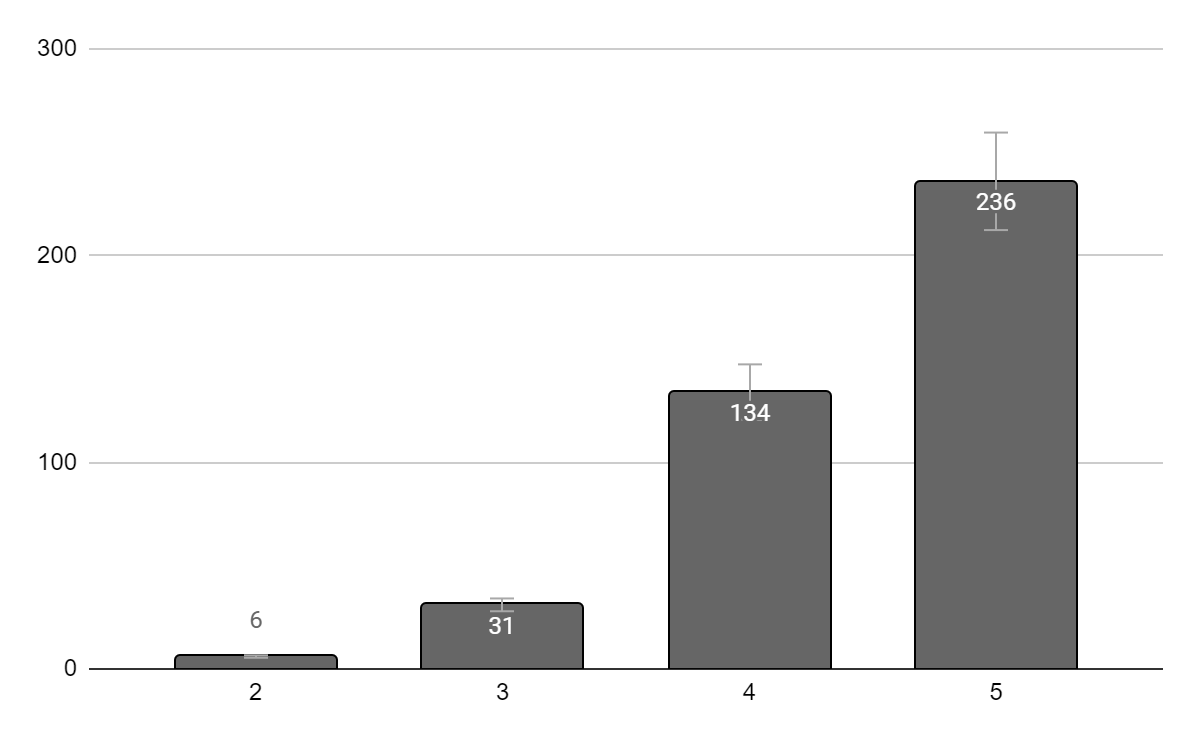
"How satisfied were you with the platforms used for the sessions (ie, Vimeo, GotoWebinar, and Facebook Live)?" Evaluation of the conference by the attendees. Information available for 408 attendees (1=not satisfied to 5=very satisfied).

At the end of the meeting, 408 attendees evaluated the conference and provided feedback in the form of a questionnaire. Questions with participant responses are presented in Figures 2 and 3. All participants reported that the conference was highly relevant to their professional practice. Additionally, using a scale of 1 to 5 (5 being extremely satisfied and 1 not satisfied at all), the majority of the participants were extremely satisfied (n=320, 78%) or very satisfied (n=83, 20%) with the event. The majority of the participants were extremely satisfied (n=236, 58%) or very satisfied (n=134, 33%) with the platforms used for the live sessions. Approximately 30% (n=122) of the attendees suggested improvements to the technological platform and onboarding of the event. Suggested improvements included schedule improvement, onboarding of the platform, times, adding more topics, increasing time for questions, and adding clinical cases. The attendees reported feeling that the event provided a novel way to disseminate medical knowledge within clinical endocrinology. Below are some comments provided by the attendees:

Very good initiative.

Thank you for continuing distance learning.

The truth is that everything turned out very well, at first it was very difficult with the use of technology, but it improved a lot over the days.

I loved the topics and the didactics of the professionals. They were very practical and of great relevance for my daily practice in the clinic. Thanks a lot!

I am very grateful to the entire organization because it gave me the opportunity to listen to true references in pediatric endocrinology and in turn in such a way that it will be very useful for my professional practice, which at the moment is in general medicine.

**Table 2:** Origin country of attendees. The number of attendees per country and the percent per country is presented. Information available for 668 attendees.

Country	Attendees, n (%)
Guatemala	69 (10.3)
Venezuela	66 (9.9)
Dominican Republic	63 (9.4)
Costa Rica	57 (8.5)
Ecuador	54 (8.1)
Peru	50 (7.5)
Uruguay	40 (6.0)
Mexico	36 (5.4)
Honduras	35 (5.2)
Argentina	33 (4.9)
USA	31 (4.6)
Bolivia	25 (3.7)
Chile	21 (3.1)
Countries with 17 or less attendees	88 (13.2)
Total	668 (100)

## Discussion

### Principal Findings

Latin America has on average 2 doctors per 1000 population, and a low number of doctor consultations per capita [13]. Additionally, there is a lack of formally trained medical professionals in the field of pediatric endocrinology in several countries in Central and South America [14]. Some countries, such as Argentina, Brazil, and Mexico, have several trained medical professionals in pediatric endocrinology; however, other countries such as Nicaragua and Bolivia have a limited number of pediatric endocrinologists, available only in urban centers [15,16]. Previous conferences focused on pediatric endocrinology and diabetes were successfully held in Central America with the support of PES, as mentioned; however, participation was limited. As mentioned by Zacharin et al [14], access to up-to-date medical education for physicians and participation in medical conferences can be challenging for many medical professionals, trainees, and students from low-income countries where expertise and formal training is lacking [14]. Central and South America include several countries where there is a need for more formal training in pediatric endocrinology. Previous initiatives by the PES, International Society for Pediatric and Adolescent Diabetes, and the European Society for Paediatric Endocrinology have been welcomed and have provided opportunities for formal training with success [14]. However, most of these initiatives have required in-person participation, which can be particularly challenging for medical professionals from low-income countries.

Here, we present the first virtual pediatric endocrine conference in low- and middle-resource countries in Central and South America and the Caribbean with the support of the PES. There were several factors that contributed to the success of the conference. The virtual platform of the meeting resulted in a larger number of participants (668 participants) compared to the previous conferences in Costa Rica (close to 50 participants) and the Dominican Republic (close to 150 participants) and offered an opportunity for attendance to health care professionals from 20 countries. Over half of the participants were pediatricians and general practitioners who do not specialize in pediatric endocrinology, and who might have not had the opportunity to attend the conference if it was only offered in person. Additionally, most of the sessions were available in Spanish, the native language for most participants. Lastly, the conference covered a wide variety of topics related to pediatric endocrinology and diabetes (Table 1) and, according to the participants, these topics were highly relevant to their professional practice. The participants were very satisfied with the event (Figure 2) and the platforms used for the live sessions (Figure 3).

The recent challenges due to the COVID-19 pandemic have resulted in most large conferences being held virtually. Virtual conferences are more accessible, more inclusive, and more affordable, and offer opportunity to the attendees to access material at their convenience. They are also less time-consuming as they do not include travel [17]. Registration for this conference was free, and there were many sessions that allowed for direct interaction between the presenters and the audience in the form of Q&A session, which is not always available in virtual conferences. Additionally, the participants were mostly satisfied with the technological and organizational aspects of the event, providing useful feedback for future virtual conferences.

A limitation to reporting our results was that attendees used different platforms to access the academic content. The objective was to facilitate access, thus generating fewer barriers to accessing knowledge at the cost of making the analysis of access to data from multiple web-based platforms more complex.

### Conclusions

Under this virtual conference, with the use of different virtual platforms, ease of access with free registration, and up-to-date technology, professionals who otherwise would not have attended international conferences were able to attend high-level scientific lectures on pediatric endocrinology and diabetes. The participants were satisfied with the conference content, which was relevant to their practice and well organized. The availability of low-cost and low–technical complexity access to digital platforms could promote the dissemination of medical education and is a complementary initiative to improve training opportunities for health care professionals from low-income and low- to middle-income countries in pediatric endocrinology and diabetes.

## Appendix

Multimedia Appendix 1 Agenda.

Multimedia Appendix 2 First Virtual Meeting.

## Abbreviations

PES: Pediatric Endocrine Society
