# Supplementary figures and images for: Bringing the Pediatric Endocrine Spanish Speaking Community Together: First Virtual Pediatric Endocrine Meeting in Low- and Middle-Income Countries in Central and South America
Source: Interact J Med Res. 2023 May 8;12:e41353. doi: 10.2196/41353 (PMC10203921; doi:10.2196/41353)

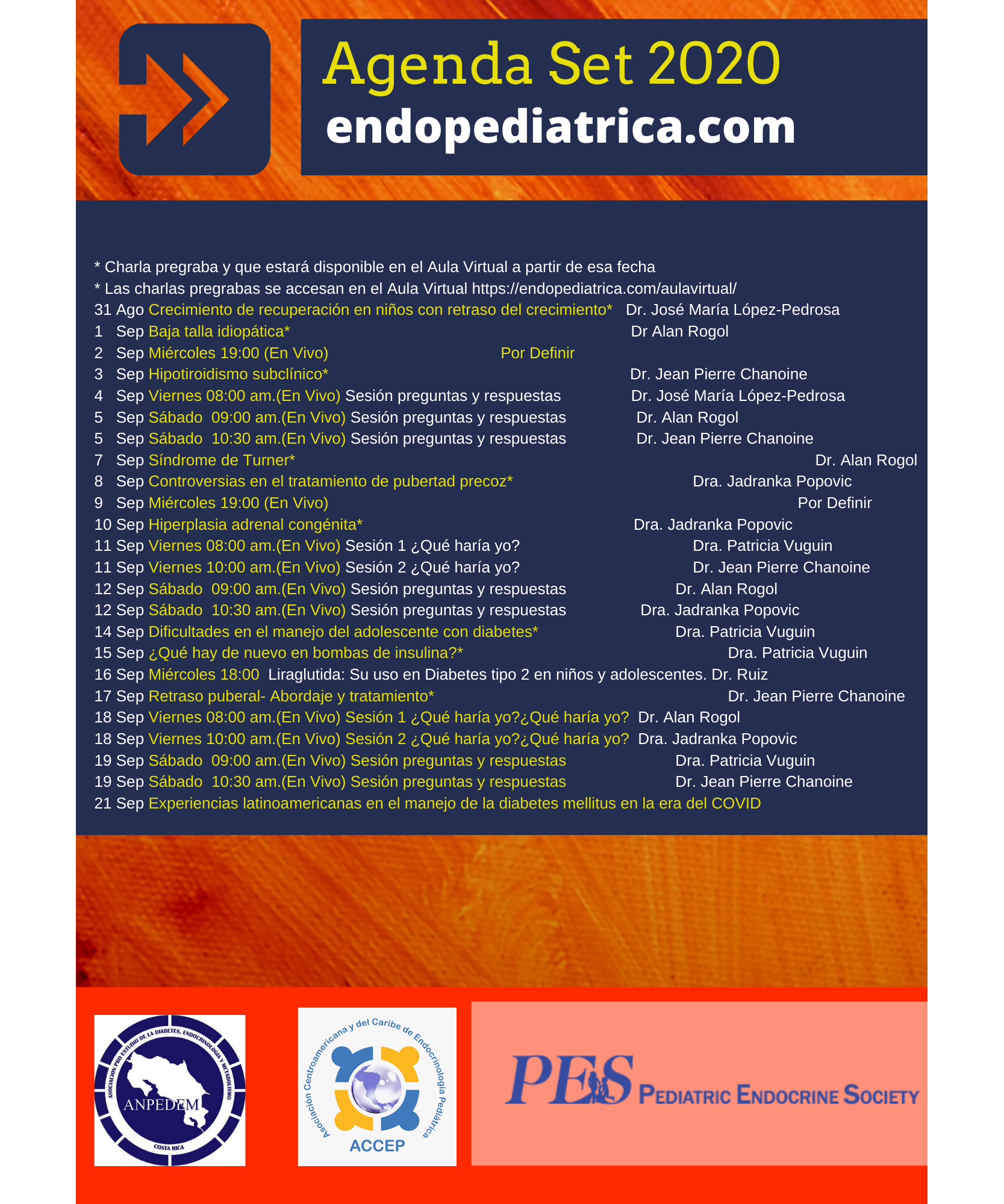

Supplement: Multimedia Appendix 1 [file ijmr_v12i1e41353_app1.png]

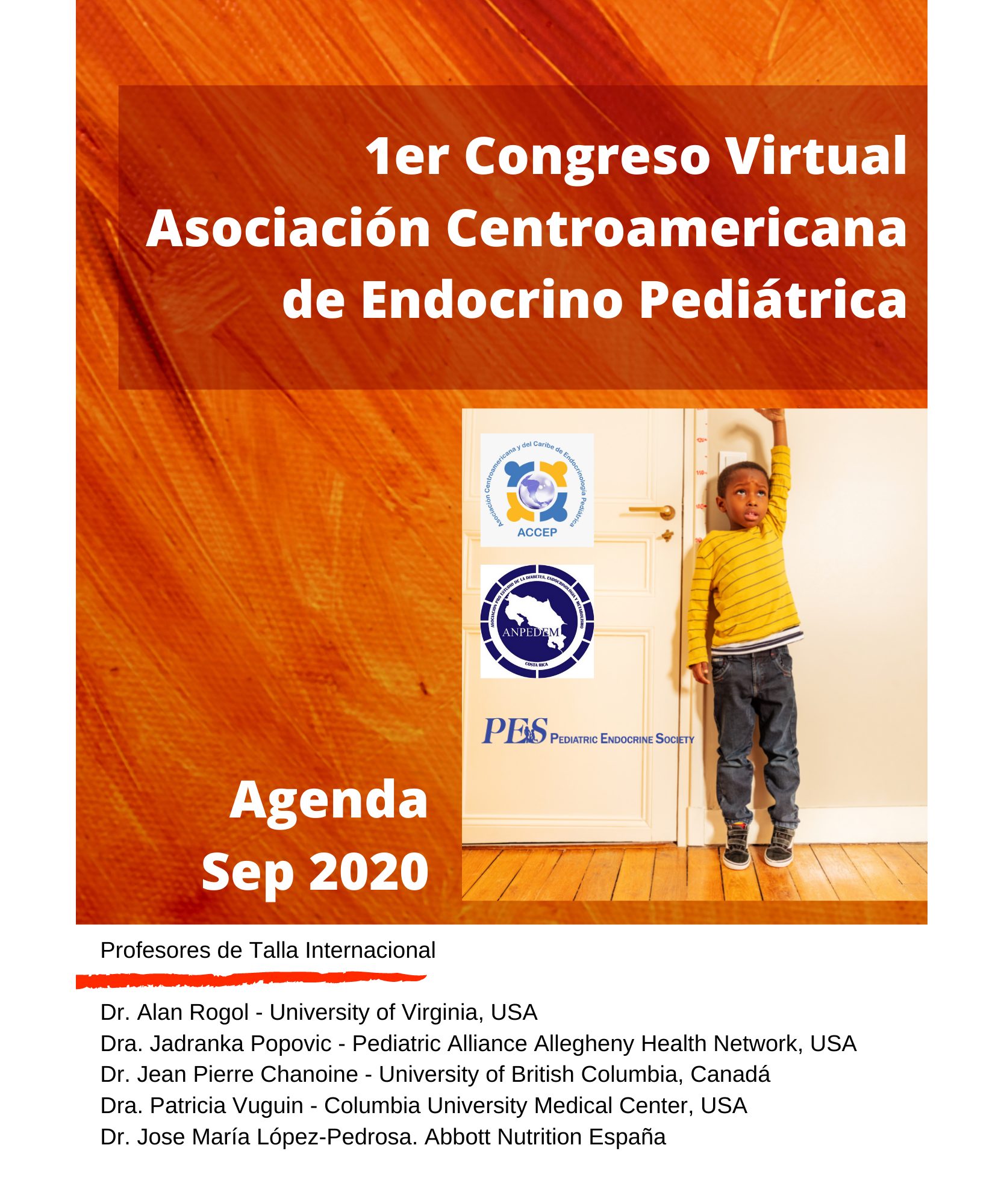

Supplement: Multimedia Appendix 2 [file ijmr_v12i1e41353_app2.png]
